# 

**Published:** 1895-12-21

**Authors:** 


### CONTENTS.

**The Proper Business of the Coroner** - 193
**School Board Certificates** - 194
**The Study of Man.**—Education in Anthropology - 195
**Medical Progress and Hospital Clinics**—
On the Differential Diagnosis of Popliteal Swellings. By Alexander Miles, M.D. - 199
Progress in Medicine.—Anæmia, Leukæmia, and Lymphadenoma—General Pathology (*continued*) - 200
Progress in Surgery.—Morbid Growths - 203
New Appliances and Things Medical - 204
**Down with the Butcher** - 196
**Notes and News** - 198
**Annotations.**—The Poisoner, the Doctor, and the Nurse—Drunk or Dying—The Chelsea Hospital - 197
**The Institutional Workshop**—
Hospital Expenditure.—The Commissariat. X.—The Servants' Table - 205
Practical Departments - 206
The Story of the Insane from Year to Year - 206
**Scraps and Gleanings** - 207

### EXTRA SUPPLEMENT.—THE NURSING MIRROR.

**News from the Nursing World.**—A Christmas Dinner—Wanted, Boots and Shirts—Training for Soldiers' Wives—King's College Hospital—Haggerston and Hoxton Nurses—Christmas at Charing Cross Hospital—An Entertainment at Wolverhampton—Queen's Nurses in Lincoln—Teachers' Home of Rest—The Peter Brough Nurses—Haverfordwest Infirmary—Lost Prope·ty—Our American Sisters—A Westminster Nurse—New Nursing Staff for Johannesburg—Short Items - xcv

**Lectures on Nursing** - xcvii

**Appointments** - xcvii

**"A National System of Notification and Registration of Sickness"** - xcviii

**St. George's Hospital** - xcviii

**Minor Appointments** - cii

**Our American Letter** - c

**Everybody's Opinion** - ci

**Notes and Queries** - cii

**A Suggestion for Uniform** - cii

**Christmas in Hospital** - xcix

